# Ultrasound biomicroscopy study of accommodative state in Smartphone abusers

**DOI:** 10.1186/s12886-022-02557-x

**Published:** 2022-08-03

**Authors:** Randa Farouk Kashif, Mohammad Ahmad Rashad, Azza Mohamed Ahmed Said, Menan Abd-El-Maksoud Rabie, Wael Adel Gomaa

**Affiliations:** 1 Ophthalmology Department, Faculty of Medicine, Ain Shams University, Cairo, Egypt; 2 Psychiatry Department, Faculty of Medicine, Ain Shams University, Cairo, Egypt

**Keywords:** Smartphone addiction, Spasm, Accommodation, Ultrasound biomicroscopy

## Abstract

**Background:**

Addiction to Smartphone usage has psychological and physical impacts. However, the state of spasm of accommodation is unclear in Smartphone abusers.

**Methods:**

We performed a cross-sectional study among adults aged 18–35 years between October 2016 and December 2018. Forty participants were Smartphone abusers according to the Smartphone addiction questionnaire, and 40 participants were non users. We measured auto refraction precycloplegia and postcycloplegia at far for all participants to evaluate the state of spasm of accommodation. We assessed the ultrasound biomicroscopy (UBM) parameters including anterior chamber angle (ACA).

**Results:**

There was a significant difference in the odds of having spasm of accommodation between Smartphone abusers compared to non-users (OR = 6.64, 95% CI = 1.73–25.47; adjusted OR = 14.63, 95% CI = 2.99–71.62). The Smartphone abusers and non-users groups had a superior ACA median of 30.45^°^ ± 8.3^°^ vs. 26.75^°^ ± 6.6^°^ (*P* = 0.04) precycloplegia at far and 31.70^°^ ± 11.8^°^ vs. 31.45^°^ ± 8.3^°^ (*P* = 0.15) postcycloplegia at far, respectively, demonstrated by the Mann-Whitney U test. There was significant higher difference in the precycloplegic nasal ACA at far in the Smartphone abusers group than the non-users group (mean precycloplegic nasal ACA difference = 3.57^°^, 95% CI = 0.76^°^ - 6.37^°^), demonstrated by the independent t test. Similarly, there was significant higher difference in the postcycloplegic nasal ACA at far (mean postcycloplegic nasal ACA difference = 4.26^°^, 95% CI = 1.33^°^ - 7.19^°^).

**Conclusions:**

Smartphone abusers are in a condition of accommodation spasm. As a result, cycloplegic refraction should be done for Smartphone abusers.

**Supplementary Information:**

The online version contains supplementary material available at 10.1186/s12886-022-02557-x.

## Background

The Smartphone is a widely used device all over the world. It has become the mainstay of communication and access to information on the internet. The number of Smartphone users has exceeded six billion worldwide in 2022. This number is expected to be 7.69 billion in 2027 [1].

Smartphone addiction is considered as the inability to control Smartphone use despite negative effects on users. Smartphone addiction is based upon Internet addiction due to similar symptoms and negative effects on users [2]. Internet and Smartphone addiction are behavioral and not substance dependent [3, 4].

Smartphone addiction has been associated with psychological and physical impacts. Psychological problems include anxiety, depression and sleep disturbance [5–10]. Physical problems include musculoskeletal problems, accidents, headache and neurological problems [11].

Narawi et al. [12] reported that after using Smartphones for 20 minutes, there was a weakness in accommodation. Similarly, Park et al. [13] reported a change of accommodative functions following 30 minutes of Smartphone usage. However, the state of spasm of accommodation in Smartphone abusers is still unknown.

The aim of the current cross sectional study is to evaluate the state of accommodation in Smartphone abusers compared to non-users and to detect any ultrasound biomicroscopy (UBM) parameters changes.

## Methods

This cross sectional study was conducted on a selected (non- randomized) sample of subjects at the department of ophthalmology, Ain –Shams University Hospitals in Cairo, between October 2016 and December 2018. Written informed consents were obtained from all participants. This study was approved by the ethical committee under study ID Number FMASU MD 43/2017, was registered on ClinicalTrials.gov Identifier NCT03389009 on 3/1/2018 and was conducted according to the principles of the Declaration of Helsinki. The study enrolled 80 eyes from 80 participants. They were divided into 2 groups according to the Smartphone questionnaire (Additional file 1) group (1): Smartphone Abusers and group (2): Smartphone non users. The inclusion criteria were healthy participants, aged between 18 and 35 years and emmetropes or myopes. The exclusion criteria were participants with hypermetropia, binocular vision abnormality, any form of tropia, premature presbyopia or previous intraocular surgery and participants taking drugs inducing spasm of accommodation.

All participants were asked about personal history, past medical history, occupational, family history and three questionnaires (Additional file 1).

1. Smartphone addiction test for Smartphone abuse assessment:
  The Smartphone addiction test is based upon Internet addiction test [14] with strong internal consistency (α = 0.93) and good test-retest reliability (*r* = 0.83) [15].
2. Beck Anxiety Inventory (BAI) for anxiety assessment:
  The BAI had high internal consistency (Cronbach’s coefficient alpha = 0.94) and was acceptably reliable over an average time lapse of 11 days (*r* = 0.67). The BAI proved better on tests of convergent and discriminant validity than did Trait Anxiety [16].
3. Beck Depression Inventory (BDI) for depression assessment:
  Beck’s study reported a Cronbach’s coefficient alpha rating of 0.92. The BDI-II positively correlated with the Hamilton Depression Rating Scale, *r* = 0.71, had a one-week test–retest reliability of *r* = 0.93 and an internal consistency α = 0.91 [17].

The questionnaires were done before the auto refraction assessment. A complete eye examination was performed and included uncorrected visual acuity, refraction by auto refractometer without cycloplegia and with cycloplegia, anterior segment slit lamp examination, fundus examination and ultrasound biomicroscopy examination (UBM) without cycloplegia and with cycloplegia. The Snellen eye chart was used to assess VA for far. The Snellen visual acuity was transformed to logMAR.

### Ultrasound biomicroscopy (UBM)

#### Procedure

Vumax 5150 machine (Sonomed, USA) was used under standard illumination with a 35-MHz probe. The procedure was done by one examiner. The participant was asked to lie in the supine position, then a local anaesthetic eye drop [4% benoxinate hydrochloride (BENOX® by E.I.P.I.Co)] was instilled and after 5 minutes an immersion cup with a smooth flanged inferior margin that fits between the lids and holds them open was used. The cup was filled with the coupling fluid and the probe was held as the transducer and a part of the probe were soaked in the coupling fluid perpendicular to the cornea but not touching it. The participant was instructed to look at a fixation point for the left eye located on the ceiling 3.0 m away, which was considered a far distance. UBM examination was done with the setting of examination sulcus to sulcus that obtained videos showing perpendicular views of the eye. The probe was then rotated 90 degrees on the same setting to get the nasal and temporal angles of the anterior chamber. To assess the angle width, the participant was asked to look in the direction opposite to the angle to be assessed. We measured the anterior chamber angle (ACA) at superior, inferior, temporal and nasal positions, trabecular ciliary process distance (TCPD), anterior chamber depth (ACD) and lens thickness. The cup was removed and a cycloplegic eye drop [1% cyclopentolate (colircusi cicloplejico® by ALCON] was instilled as a drop every 10 minutes for three times (Total time: 30 minutes). Then UBM examination was repeated after cycloplegia with the same settings and the same parameters were measured [18].

#### Refractive data analysis

With the spasm of accommodation as the primary outcome, the refractive data for each participant were first converted to matrix form according to Long’s dioptric power matrix [19] as follows:

$$\mathrm{F}=\left(\begin{array}{cc}f11& f12\\ {}f21& f22\end{array}\right)\ as$$

$$f_{11}=\mathrm S+\mathrm C\;\sin^{2}\;\mathrm A,$$

$$f_{12}=f_{21}=-\mathrm C\;\mathrm s\mathrm i\mathrm n\;\mathrm A\;\mathrm c\mathrm o\mathrm s\;\mathrm A$$

$${F}_{22}=\mathrm{S}+\mathrm{C}\ {\cos}^2\ \mathrm{A}$$

We calculated the precycloplegic *f*_11,_ *f*_12_ and *f*_22_ and postcycloplegic *f*_11,_ *f*_12_ and *f*_(22)_

Then we measured the difference between postcyloplegic and precycloplegic in Long’s dioptric form.

$${f}_{11\_}\mathrm{difference}={f}_{11\_}\mathrm{post}-{f}_{11\_}\mathrm{pre}$$

$${f}_{12\_}\mathrm{difference}={f}_{12\_}\mathrm{post}-{f}_{12\_}\mathrm{pre}$$

$${f}_{22\_}\mathrm{difference}={f}_{22\_}\mathrm{post}-{f}_{22\_}\mathrm{pre}$$

Then we transformed the differences back to the conventional notation using Keating’s procedure [20] as follows:

The trace *t* = *ƒ*_11_ + *ƒ*_12_ and determinant *d* = *ƒ*_11_*ƒ*_22_ – *ƒ*_12_*ƒ*_21_. Then, the cylinder was calculated using *C* = $\sqrt{t^2-4d}$, the sphere with S = (t-C)/2 and the axis by tan A = (S- *ƒ*_11_)/*ƒ*_12_.

According to the results of the difference for each participant, the participant was considered to have a spasm of accommodation in either the sphere difference between postcyloplegic and precycloplegic was > 1.00 diopters or the cylinder difference between postcycloplegia and prescycloplegia was ≥0.5 diopters.

For refractive data as a secondary outcome, the same Long’s dioptric power matrix was used. The precycloplegic *f*_11, _*f*_12 _and *f*_22 _and postcycloplegic *f*_11, _*f*_12_ and *f*_22 _were calculated for all participants. The means of precycloplegic *f*_11, _*f*_12 _and *f*_22 _and postcycloplegic *f*_11, _*f*_12 _and *f*_22 _were algebraically obtained by adding and averaging vectorial components [21].

### Statistical analysis

The data were collected from the right eye and analyzed using SPSS (IBM Statistical Analysis) version 22.0. The Chi square test was used for nominal data. The normality of continuous data was checked by the Shapiro-Wilk test of normality. The independent t test was used for continuous data fulfilling the assumptions including normality, while Mann Whitney U test was used for continuous data not fulfilling the assumptions. The primary outcome, spasm of accommodation, was assessed by odds ratio and was adjusted for age difference between the 2 groups using logistic regression. As the assumptions of parametric multivariate statistics were not met for the refractive data, the ANOVA type statistic of nonparametric multivariate using the R package npmv version 2.4.0 was done [22]. Statistical significance was accepted at *P* < 0.05.

## Results

Our study consisted of 80 eyes from 80 participants aged between 18 to 35 years old (38 females and 42 males) and were divided into 2 groups according to the Smartphone questionnaire:

- Group (1): 40 Smartphone abuser participants .
- Group (2): 40 Smartphone non user participants

I Demographic analysis

The median age of Smartphone abusers was 26 years (IQR = 7) compared to 31 years (IQR = 5.5) for non-users group. In the Smartphone abusers group, 16 of 40 participants (40%) were females and in the non-users group, 22 of 40 participants (55%) were females. In the Smartphone abusers group, 9 (23%) participants had a family history of diabetes mellitus, but in the non-users group 13 (32.5%) participants had a family history of diabetes mellitus. As regards hypertension, in Smartphone abusers group 8 (20%) participants had a family history of hypertension while in non-users group 5 (12.5%) participants had family history of hypertension. The Mann-Whitney U test revealed significant difference in age between the Smartphone abusers group and the non-users group *U* = 374.00 (*Z* = − 4.117), *P* < 0.001, *r* = 0.46. The Chi-square test of independence was conducted to compare sex, diabetes family history and hypertension family history in the Smartphone abusers group and the non-users group. There was a statistically insignificant difference in sex between the Smartphone abusers group and the non-users group *x*^*2*^ (1, *N* = 80) = 1.80, *P* = 0.18. Similarly, there was an insignificant difference in diabetes family history and hypertension family history. Details of demographic characteristics are shown in Table 1.

**Table 1:** Demographic characteristics

	Smartphone abusers (***n*** = 40)	Non users (***n*** = 40)	Statistical test	***P***-value
**Age years** **(Median** **±** **IQR)**	26 ± 7	31 ± 5.5	374.00^a^	< 0.001*
**Sex (male) No. (%)**	24 (60%)	18 (45%)	1.80^b^	0.18
**FH of DM (with) No. (%)**	9 (23%)	13 (32.5%)	1.00^b^	0.32
**FH of HTN (with) No. (%)**	8 (20%)	5 (12.5%)	0.83^b^	0.36

*IQR* Interquartile range

^a^Mann-Whitney U test was conducted.

^b^Chi-square test of independence was conducted.

* Statistically significant *P* < 0.05

II. Psychological analysis

For the Smartphone abuse questionnaire score for the abusers group, the mean Smartphone addiction score was 73.35 ± 8.85. The median for the Smartphone abuse category was mild and the mode was mild. The mean Smartphone use hours was 6.60 ± 1.81.

The Mann-Whitney U test revealed an insignificant difference in anxiety questionnaire score between the Smartphone abusers group and the non-users group *U* = 721.00 (*Z* = − 0.76), *P* = 0.45, *r* = − 0.08. The median anxiety score was 4 (IQR = 6.00; mean rank = 42.48) for the Smartphone abusers group compared to 4 (IQR = 4.80; mean rank = 38.53) for the non-users group.

The Mann-Whitney U test revealed an insignificant difference in depression questionnaire score between the Smartphone abusers group and the non-users group *U* = 662.00 (*Z* = − 1.33), *P* = 0.19, *r* = − 0.15. The median depression score was 6.5 (IQR = 6.00; mean rank = 43.94) for the Smartphone abusers group compared to 4.5 (IQR = 9.80; mean rank = 37.06) for the non-users group (Table 2).

**Table 2:** Anxiety and depression scores comparison

	Smartphone abusers (***n*** = 40)	Non users (***n*** = 40)	Mann-Whitney test	***P***-value
**Anxiety questionnaire score (Median** **±** **IQR)**	4 ± 6	4 ± 4.8	721	0.45
**Depression questionnaire score (Median** **±** **IQR)**	6.5 ± 6	4.50± 9.8	662	0.19

*IQR* Interquartile range

Mann-Whitney U test was conducted

Significance level *P* < 0.05

III. Spasm of accommodation as the primary outcome

In the Smartphone abusers group, 37 (92.5%) participants were spastic and 3 (7.5%) participants were non-spastic and in the non-users group, 26 (65.0%) participants were spastic and 14 (35.0%) participants were non-spastic.

The Chi-square test of independence was conducted to compare spasm of accommodation in the Smartphone abusers group and the non-users group. There was a statistical significant difference in spasm of accommodation between the Smartphone abusers group and the non-users group *x*^*2*^ (1, *N* = 80) = 9.04, *P* = 0.003 (Table 3).

**Table 3:** Spasm accommodation comparison

Spasm of Accommodation	Smartphone abusers (***n*** = 40)	Non users (***n*** = 40)	Chi-square	***P***-value
**Number of Spastic (%)**	37 (92.5%)	26 (65.0%)	9.04	0.003*
**Number of Non spastic (%)**	3 (7.5%)	14 (35.0%)		

Chi-square test of independence was conducted, * Statistically significant *P* < 0.05

Smartphone abusers were 6.64 times more likely to have spasm of accommodation than non-users (Odds ratio = 6.64, 95% CI = 1.73–25.47, *P* = 0.006). On controlling for age difference between the 2 groups, the adjusted odds ratio was 14.63 (Adjusted odds ratio = 14.63, 95% CI = 2.99–71.62, *P* = 0.001).

IV. Refractive data analysis

The median unaided distance logMAR visual acuity of Smartphone abusers was 0.30 (IQR = 0.79) compared to 0.00 (IQR = 0.30) for the non-users group, while all participants achieved the best distance logMAR visual acuity 0.00. Tables 4 and 5 summarize the descriptive data of refractive errors in the Smartphone abusers group and the non-users group using Long’s dioptric power matrix.

**Table 4:** Mean vectors of the precycloplegic, postcycloplegic and difference of refraction in Smartphone abusers

Smartphone Abusers	Matrix formalism (***n*** = 40)			Standard notation (***n*** = 40)		
	*f*_11_	*f*_12_	*f*_22_	Sphere (dioptres)	Cylinder (dioptres)	Axis (degree)
**Precycloplegic refraction**	−1.61	0.05	−1.98	−1.98	0.38	97^°^
**Postcycloplegic refraction**	−0.38	0.03	−0.64	− 0.64	0.26	96^°^
**Difference of postcycloplegic and precycloplegic**	1.23	−0.02	1.34	1.22	0.11	7^°^

*f*_11,_ *f*_12,_ *f*_22_ are Long’s dioptric power matrix forms

**Table 5:** Mean vectors of the precycloplegic, postcycloplegic and difference of refraction in non users

Non users	Matrix formalism (***n*** = 40)			Standard notation (***n*** = 40)		
	*f*_11_	*f*_12_	*f*_22_	Sphere (dioptres)	Cylinder (dioptres)	Axis (degree)
**Precycloplegic refraction**	−0.79	0.05	−0.89	− 0.90	0.14	111^°^
**Postcycloplegic refraction**	0.12	0.05	0.03	0.00	0.12	113^°^
**Difference of postcycloplegic and precycloplegic**	0.90	−0.001	0.92	0.90	0.01	4^°^

*f*_11,_ *f*_12,_ *f*_22_ are Long’s dioptric power matrix forms

Nonparametric multivariate analysis was used for refractive data using the Long’s dioptric power matrix.

The ANOVA type statistics of nonparametric multivariate analysis of precycloplegic refraction revealed that there was statistically significant difference in the mean vector between the Smartphone abusers group and the non-users group (F (2.21, 172.46) =5.043, *P* = 0.006).

The ANOVA type statistic of nonparametric multivariate analysis of postcycloplegic refraction revealed that there was statistically insignificant difference in the mean vector between the Smartphone abusers group and the non-users group (F (2.11, 164.19) = 0.27, *P* = 0.77).

The ANOVA type statistic of nonparametric multivariate analysis of the difference between postcycloplegic and precycloplegic refraction revealed that there was a statistically significant border line difference in the mean vector between the Smartphone abusers group and the non user group (F (2.23, 174.20) = 2.86, *P* = 0.054).

XXII. Ultrasound biomicroscopic (UBM) parameters

The median precycloplegic superior anterior chamber angle (ACA) at far was 30.45^°^ (IQR = 8.3^°^; mean rank = 45.94) for Smartphone abusers group compared to 26.75^°^ (IQR = 6.6^°^; mean rank = 35.06) for the non users group. The Mann-Whitney U test revealed a significant difference between the Smartphone abusers group and the non-users group for precycloplegic superior ACA at far *U* = 582.50 (*Z* = − 2.09), *P* = 0.04, *r* = 0.23. However, the Mann-Whitney U test revealed an insignificance difference between the Smartphone abusers group and the non-users group for postcycloplegic superior ACA at far *U* = 651.50 (*Z* = − 1.43), *P* = 0.15, *r* = 0.16. The median postcycloplegic superior ACA at far was 31.70^°^ (IQR = 11.8^°^; mean rank = 44.21) for the Smartphone abusers group, compared to 31.45^°^ (IQR = 8.3^°^; mean rank = 36.79) for the non-users group. The mean difference of the superior ACA difference between postcycloplegia and precycloplegia at far between the Smartphone abusers group and the non-users group was not statistically significant (mean difference = − 0.37^°^ with a 95% confidence interval ranging from − 2.89^°^ to 2.15^°^, *P* = 0.77).

There was significant higher difference in the precycloplegic nasal ACA at far in the Smartphone abusers group than the non-users group (mean precycloplegic nasal ACA difference = 3.57^°^ with a 95% confidence interval ranging from 0.76^°^ to 6.37^°^, *P* = 0.01). Similarly, there was significant higher difference in the postcycloplegic nasal ACA at far (mean postcycloplegic nasal ACA difference = 4.26^°^ with a 95% confidence interval ranging from 1.33^°^ to 7.19^°^, *P* = 0.005). However, there was no significant difference in the nasal ACA difference between postcycloplegia and precycloplegia at far (mean difference = 0.70^°^ with a 95% confidence interval ranging from − 2.44^°^ to 3.84^°^, *P* = 0.66).

There was significant lower precycloplegic lens thickness at far in the Smartphone abusers group (mean precycloplegic lens thickness difference = − 0.22 mm with a 95% confidence interval ranging from − 0.33 mm to − 0.10 mm, *P* < 0.001) Similarly, there was significant lower postcycloplegic lens thickness by the Mann-Whitney U test (*U* = 496.50 (*Z* = − 2.92), *P* = 0.003, *r* = 0.33). The median of the postcycloplegic lens thickness at far was 3.27 mm (IQR = 0.41 mm; mean rank = 32.91) for Smartphone abusers group, compared to 3.50 mm (IQR = 0.31 mm; mean rank = 48.09) for the non users group. However, there was no significant difference in the lens thickness difference between postcycloplegia and precycloplegia at far from the Mann-Whitney U test (*U* = 678.50 (*Z* = − 1.17), *P* = 0.24, *r* = 0.13). The median lens thickness difference between postcycloplegia and precycloplegia at far was − 0.07 mm (IQR = 0.18 mm; mean rank = 43.54) for the Smartphone abusers group, compared to − 0.11 mm (IQR = 0.15 mm; mean rank = 37.46) for the non-users group.

The other parameters including anterior chamber depth (ACD), trabecular ciliary process distance, temporal ACA and inferior ACA showed no significant difference in either precycloplegia, postcyloplegia or difference between postcyloplegia and precycloplegia at far (Supplementary Table 1, Supplementary Table 2).

VI. Correlation between Smartphones spent hours, spasm of accommodation and Ultrasound biomicroscopic (UBM) parameters

A point-biserial correlation test was conducted to determine the relationship between Smartphones spent hours and spasm of accommodation. There was a very week insignificant correlation between Smartphones spent hours and spasm of accommodation (*r*_*pb*_ = 0.04, *n* = 40, *P* = 0.79).

Spearman’s correlation test was conducted to determine the relationship between Smartphone spent hours and UBM parameters and revealed insignificant correlation (Supplementary Table 3, Supplementary Table 4).

## Discussion

After correcting for the age difference between the two groups, Smartphone abusers were 14.63 times more likely to experience spasm of accommodation than non-users. The non-adjusted odds ratio of spasm of accommodation was 6.64. This is an expected difference in age between the Smartphone abusers and non-users. The tendency to use Smartphones is in a younger age group. This difference in age was corrected by logistic regression which unmasked the true difference between the 2 groups. This accommodation spasm result is consistent with that of Narawi et al. [12]. However, they compared the before and after effects of using a Smartphone for 20 minutes. In our study we subtracted precycloplegic refraction from postcycloplegic and we compared the result with threshold 1 for the sphere and threshold 0.5 for the cylinder while. Narawi et al. measured the amplitude of accommodation.

In the current study, the anxiety and depression scores had non-significant differences between the 2 groups. Therefore, the influence of the limbic system on accommodation was similar in the Smartphone abuser group and the non user group. Khalid et al. [23] state that there is an association between pseudomyopia and anxiety as a result of excessive accommodation.

In analyzing refractive error data, the correct way of analyzing refractive data was utilized by transforming the data into Long’s dioptric power matrix [19–21]. A multivariate non-parametric test was used as the refractive error data did not fulfil the assumption for the parametric test [22]. While there was a statistically significant difference in precycloplegic refraction, the postcycloplegic refraction showed non-statistically significant difference between the 2 groups. This reveals pseudomyopia and excessive accommodation in Smartphone abusers. In Liang et al. Study [24], there was a near work-induced transient myopia for reading with a mobile phone and reading with text for 40 minutes. But, there was no significant difference between reading with mobile phone and reading with text. Our study did not only show a transient myopia but a state of accommodation spasm. This difference is explained by the hours spent by Smartphone abusers, not just 40 minutes.

In the present study, the superior anterior chamber angle (ACA) showed statistically significant difference in precycloplegia between the Smartphone abusers and the non-users while the postcyloplegic superior ACA did not vary significantly. This indicates spasm of accommodation completely relieved by complete cycloplegia. Moreover, both the nasal ACA and the lens thickness varied significantly in precycloplegia and postcycloplegia between the two groups. This might indicate partial relaxation of ciliary muscle or incomplete cycloplegia. However, temporal ACA, inferior ACA, trabecular ciliary process distance and anterior chamber depth (ACD) were not affected. In Dominiguez-Vicent et al. study [25], they found no significant ACD change during accommodation using Dual Scheimpflug and a Placido disc. However, the ACA, at the superior, temporal and inferior positions, varied significantly during accommodation. Our results are in agreement with those of Dominiguez-Vicent et al. in ACD, superior ACA and inferior ACA and differ in temporal ACA, inferior ACA and nasal ACA. This might be due to different instruments used to measure the ACA. In Marchini et al. study [26], ACD was not affected during accommodation with monofocal intraocular lens, assessed by the UBM 840 system and the HiScan system. These authors reported significant variation in vertical ACA measured by the Hiscan system and horizontal ACA measured by the UBM 840 system. Although the authors’ objective was to detect changes during accommodation in eyes with a monofocal lens, those findings partially agree with our results. The difference could be due to different sample populations. Marchini et al. studied old adults while we included young adults in our study. We studied the Smartphone abusers with phakic eyes whereas Marchini et al. comprised pseudophakic eyes.

One of the limitations of our study is the lack of repeated measurements in ultrasound biomicroscopy UBM. Either inter-observer reliability or intra-observer reliability is needed to increase the reliability of measurements.

## Conclusions

There is a state of spasm of accommodation in Smartphone abusers. Therefore, the refraction for Smartphone Abusers should be routinely done under complete cycloplegia.

## Supplementary Information

**Additional file 1.** Questionnaires English version. Arabic Questionnaires which include Smartphone addiction test for Smartphone abuse assessment, Beck Anxiety Inventory for anxiety assessment and Beck Depression Inventory for depression assessment.

**Additional file 2: Supplementary Table 1.** Description of data. Anterior chamber angle (ACA) in Smartphone abusers and nonusers.

**Additional file 3: Supplementary Table 2.** Anterior chamber depth (ACD), lens thickness and trabecular ciliary process distance (TCPD) in Smartphone abusers and nonusers.

**Additional file 4: Supplementary Table 3.** Correlation between Anterior chamber angle (ACA), spasm of accommodation and Smartphones spent hours.

**Additional file 5: Supplementary Table 4.** Correlation between Anterior chamber depth (ACD), lens thickness, trabecular ciliary process distance (TCPD) and Smartphones spent hours.

## Data Availability

The dataset supporting the conclusions of this article is available in the figshare repository, https://figshare.com/articles/dataset/DATASET_Doctorat_paper_2022_march_14_2_xlsx/19375331

## Abbreviations

UBM: Ultrasound biomicroscopy
ACA: Anterior chamber angle
ACD: Anterior chamber depth
TCPD: Trabecular ciliary process distance
BAI: Beck Anxiety Inventory
BDI: Beck Depression Inventory
