# Supplementary material for: Ultrasound biomicroscopy study of accommodative state in Smartphone abusers
Source: BMC Ophthalmol. 2022 Aug 3;22:330. doi: 10.1186/s12886-022-02557-x (PMC9347154; doi:10.1186/s12886-022-02557-x)
Supplement: Supplementary file 1 — Additional file 1. Questionnaires English version. Arabic Questionnaires which include Smartphone addiction test for Smartphone abuse assessment, Beck Anxiety Inventory for anxiety assessment and Beck Depression Inventory for depression assessment. [file 12886_2022_2557_MOESM1_ESM.doc]

**Beck Anxiety Inventory**

|  |  | | **Not at all 0** | | **Mildly but it did not bother me much. 1** | **Moderately – it wasn’t pleasant at times 2** | | **Severely - it bothered me a lot 3** |
| --- | --- | --- | --- | --- | --- | --- | --- | --- |
|  | **Numbness or tingling** | |  | |  |  | |  |
|  | **Feeling hot** | |  | |  |  | |  |
|  | **Wobbliness in legs** | |  | |  |  | |  |
|  | **Unable to relax** | |  | |  |  | |  |
|  | **Fear of worst happening** | |  | |  |  | |  |
|  | **Dizzy or lightheaded** | |  | |  |  | |  |
|  | **Hear pounding / racing** | |  | |  |  | |  |
|  | **Unsteady** | |  | |  |  | |  |
|  | **Terrified or afraid** | |  | |  |  | |  |
|  | **Nervous** | |  | |  |  | |  |
|  | **Feeling of choking** | |  | |  |  | |  |
|  | **Hands trembling** | |  | |  |  | |  |
|  | **Shaky / unsteady** | |  | |  |  | |  |
|  | **Fear of losing control** | |  | |  |  | |  |
|  | **Difficulty in breathing** | |  | |  |  | |  |
|  | **Fear of dying** | |  | |  |  | |  |
|  | **Scared** | |  | |  |  | |  |
|  | **Indigestion** | |  | |  |  | |  |
|  | **Faint / lightheaded** | |  | |  |  | |  |
|  | **Face flushed** | |  | |  |  | |  |
|  | **Hot / cold sweats** | |  | |  |  | |  |
| **0-7 🡪 minimal** | | **8-15 🡪 mild** | | **16-25 🡪 moderate** | | | **26-63 🡪 severe** | |

**Beck Depression Inventory**

**This depression inventory can be self-scored. The scoring scale is at the end of the questionnaire.**

**1.**

**0 I do not feel sad.**

**1 I feel sad**

**2 I am sad all the time and I can't snap out of it.**

**3 I am so sad and unhappy that I can't stand it.**

**2.**

**0 I am not particularly discouraged about the future.**

**1 I feel discouraged about the future.**

**2 I feel I have nothing to look forward to.**

**3 I feel the future is hopeless and that things cannot improve.**

**3.**

**0 I do not feel like a failure.**

**1 I feel I have failed more than the average person.**

**2 As I look back on my life, all I can see is a lot of failures.**

**3 I feel I am a complete failure as a person.**

**4.**

**0 I get as much satisfaction out of things as I used to.**

**1 I don't enjoy things the way I used to.**

**2 I don't get real satisfaction out of anything anymore.**

**3 I am dissatisfied or bored with everything.**

**5.**

**0 I don't feel particularly guilty**

**1 I feel guilty a good part of the time.**

**2 I feel quite guilty most of the time.**

**3 I feel guilty all of the time.**

**6.**

**0 I don't feel I am being punished.**

**1 I feel I may be punished.**

**2 I expect to be punished.**

**3 I feel I am being punished.**

**7.**

**0 I don't feel disappointed in myself.**

**1 I am disappointed in myself.**

**2 I am disgusted with myself.**

**3 I hate myself.**

**8.**

**0 I don't feel I am any worse than anybody else.**

**1 I am critical of myself for my weaknesses or mistakes.**

**2 I blame myself all the time for my faults.**

**3 I blame myself for everything bad that happens.**

**9.**

**0 I don't have any thoughts of killing myself.**

**1 I have thoughts of killing myself, but I would not carry them out.**

**2 I would like to kill myself.**

**3 I would kill myself if I had the chance.**

**10.**

**0 I don't cry any more than usual.**

**1 I cry more now than I used to.**

**2 I cry all the time now.**

**3 I used to be able to cry, but now I can't cry even though I want to.**

**11.**

**0 I am no more irritated by things than I ever was.**

**1 I am slightly more irritated now than usual.**

**2 I am quite annoyed or irritated a good deal of the time.**

**3 I feel irritated all the time.**

**12.**

**0 I have not lost interest in other people.**

**1 I am less interested in other people than I used to be.**

**2 I have lost most of my interest in other people.**

**3 I have lost all of my interest in other people.**

**13.**

**0 I make decisions about as well as I ever could.**

**1 I put off making decisions more than I used to.**

**2 I have greater difficulty in making decisions more than I used to.**

**3 I can't make decisions at all anymore.**

**14.**

**0 I don't feel that I look any worse than I used to.**

**1 I am worried that I am looking old or unattractive.**

**2 I feel there are permanent changes in my appearance that make me look unattractive**

**3 I believe that I look ugly.**

**15.**

**0 I can work about as well as before.**

**1 It takes an extra effort to get started at doing something.**

**2 I have to push myself very hard to do anything.**

**3 I can't do any work at all.**

**16.**

**0 I can sleep as well as usual.**

**1 I don't sleep as well as I used to.**

**2 I wake up 1-2 hours earlier than usual and find it hard to get back to sleep.**

**3 I wake up several hours earlier than I used to and cannot get back to sleep.**

**17.**

**0 I don't get more tired than usual.**

**1 I get tired more easily than I used to.**

**2 I get tired from doing almost anything.**

**3 I am too tired to do anything.**

**18.**

**0 My appetite is no worse than usual.**

**1 My appetite is not as good as it used to be.**

**2 My appetite is much worse now.**

**3 I have no appetite at all anymore.**

**19.**

**0 I haven't lost much weight, if any, lately.**

**1 I have lost more than five pounds.**

**2 I have lost more than ten pounds.**

**3 I have lost more than fifteen pounds.**

**20.**

**0 I am no more worried about my health than usual.**

**1 I am worried about physical problems like aches, pains, upset stomach, or constipation.**

**2 I am very worried about physical problems and it's hard to think of much else.**

**3 I am so worried about my physical problems that I cannot think of anything else.**

**21.**

**0 I have not noticed any recent change in my interest in sex.**

**1 I am less interested in sex than I used to be. 2 I have almost no interest in sex.**

**3 I have lost interest in sex completely.**

**0-13 🡪 minimal**

**14-19 🡪 mild**

**20-28 🡪 moderate**

**29-63 🡪 severe**

**Smartphone Addiction Test**

**Answer the following questions by using this scale:**

**0 = Not applicable, 1 = Rarely, 2 = Occasionally, 3 = Frequently, 4 = Often, 5 = Always**

|  | **0** | **1** | **2** | **3** | **4** | **5** |
| --- | --- | --- | --- | --- | --- | --- |
| **1- How often do you find that you use your Smartphone longer than you intended?** |  |  |  |  |  |  |
| **2. How often do you neglect household chores to spend more time on your Smartphone?** |  |  |  |  |  |  |
| **3. How often do you prefer the excitement of the Smartphone to intimacy with your partner?** |  |  |  |  |  |  |
| **4. How often do you form new relationships with fellow Smartphone users?** |  |  |  |  |  |  |
| **5. How often do others in your life complain to you about the amount of time you spend on your Smartphone?** |  |  |  |  |  |  |
| **6. How often do your grades or school work suffer because of the amount of time you spend on your Smartphone?** |  |  |  |  |  |  |
| **7. How often do you check your Smartphone before something else that you need to do?** |  |  |  |  |  |  |
| **8. How often does your job performance or productivity suffer because of your Smartphone?** |  |  |  |  |  |  |
| **9. How often do you become defensive or secretive when anyone asks you what you do with your Smartphone?** |  |  |  |  |  |  |
| **10. How often do you block out disturbing thoughts about your life with soothing thoughts of your Smartphone?** |  |  |  |  |  |  |
| **11. How often do you find yourself anticipating when you will use your Smartphone again?** |  |  |  |  |  |  |
| **12. How often do you fear that life without your Smartphone would be boring, empty, and joyless?** |  |  |  |  |  |  |
| **13. How often do you snap, yell, or act annoyed if someone bothers you while you are using your Smartphone?** |  |  |  |  |  |  |
| **14. How often do you lose sleep due to your Smartphone?** |  |  |  |  |  |  |
| **15. How often do you feel preoccupied with your Smartphone when it is far away, or fantasize about using it?** |  |  |  |  |  |  |
| **16. How often do you find yourself saying "just a few more minutes" when you are using your Smartphone?** |  |  |  |  |  |  |
| **17. How often do you try to cut down the amount of time you spend on your Smartphone and fail?** |  |  |  |  |  |  |
| **18. How often do you try to hide how long you've been using your Smartphone?** |  |  |  |  |  |  |
| **19. How often do you choose to spend more time on your Smartphone over going out with others?** |  |  |  |  |  |  |
| **20. How often do you feel depressed, moody, or nervous when you are not using your Smartphone, which goes away once you use it?** |  |  |  |  |  |  |

**Scoring:**

**0 – 30 = Normal level of Smartphone usage**

**31 – 49 = Mild level of Smartphone addiction**

**50 – 79 = Moderate level of Smartphone addiction**

**80 – 100 = Severe level of Smartphone addiction**
