# Supplementary material for: Ultrasound biomicroscopy study of accommodative state in Smartphone abusers
Source: BMC Ophthalmol. 2022 Aug 3;22:330. doi: 10.1186/s12886-022-02557-x (PMC9347154; doi:10.1186/s12886-022-02557-x)
Supplement: Supplementary file 2 — Additional file 2: Supplementary Table 1. Description of data. Anterior chamber angle (ACA) in Smartphone abusers and nonusers. [file 12886_2022_2557_MOESM2_ESM.doc]

**Supplementary Table 1** Anterior chamber angle (ACA) in Smartphone abusers and nonusers

|  | **Smartphone Abusers**  **(n = 40)** | **Non users**  **(n = 40)** | **Statistical test** | ***P*-value** |
| --- | --- | --- | --- | --- |
| **Superior ACA (degree)** |  |  |  |  |
| Pre (median + IQR) | 30.45 + 8.3 | 26.75 + 6.6 | 582.50† | 0.04* |
| Post(median + IQR) | 31.70 + 11.8 | 31.45 + 8.3 | 651.50† | 0.15 |
| Difference (mean + SD) | 3.88 + 4.94 | 4.25 + 6.30 | -0.29‡ | 0.77 |
| **Inferior ACA (degree)** |  |  |  |  |
| Pre (mean + SD) | 29.65 + 5.18 | 27.90 + 5.62 | 1.44‡ | 0.15 |
| Post (mean + SD) | 34.05 + 6.23 | 33.03 + 7.62 | 0.65‡ | 0.52 |
| Difference (mean + SD) | 4.40 + 6.65 | 5.13 + 8.07 | -0.44‡ | 0.66 |
| **Temporal ACA (degree)** |  |  |  |  |
| Pre (mean + SD) | 30.91 + 6.48 | 28.86 + 7.13 | 1.34‡ | 0.18 |
| Post (median + IQR) | 31.55 + 11.6 | 32.05 + 39.76 | 770.50† | 0.78 |
| Difference (mean + SD) | 2.35 + 7.63 | 3.04 + 7.16 | -0.42‡ | 0.68 |
| **Nasal ACA (degree)** |  |  |  |  |
| Pre (mean + SD) | 30.40 + 6.34 | 26.84 + 5.60 | 2.53‡ | 0.01* |
| Post (mean + SD) | 33.21 + 7.44 | 28.95 + 5.60 | 2.89‡ | 0.005* |
| Difference (mean + SD) | 2.81 + 7.12 | 2.12 + 7.00 | 0.44‡ | 0.66 |

*ACA* Anterior chamber angle; *IQR* Interquartile range

† Mann- Whitney U test was conducted

‡ Independent- samples t test was conducted

* Statistically significant *P* < 0.05
