# Supplementary material for: Ultrasound biomicroscopy study of accommodative state in Smartphone abusers
Source: BMC Ophthalmol. 2022 Aug 3;22:330. doi: 10.1186/s12886-022-02557-x (PMC9347154; doi:10.1186/s12886-022-02557-x)
Supplement: Supplementary file 3 — Additional file 3: Supplementary Table 2. Anterior chamber depth (ACD), lens thickness and trabecular ciliary process distance (TCPD) in Smartphone abusers and nonusers. [file 12886_2022_2557_MOESM3_ESM.doc]

**Supplementary Table 2 Anterior chamber depth (ACD), lens thickness and trabecular ciliary process distance (TCPD) in Smartphone abusers and nonusers**

|  | **Smartphone Abusers (n = 40)** | **Non users (n = 40)** | **Statistical test** | ***P*-value** |
| --- | --- | --- | --- | --- |
| **TCPD (mm)** |  |  |  |  |
| Pre (median + IQR) | 0.88 + 0.17 | 0.86 + 0.15 | 667.50† | 0.20 |
| Post (mean + SD) | 0.89 + 0.14 | 0.87 + 0.10 | 0.79‡ | 0.43 |
| Difference (mean + SD) | -0.01 + 0.16 | 0.02 + 0.10 | -0.97‡ | 0.33 |
| **ACD (mm)** |  |  |  |  |
| Pre (mean + SD) | 3.07 + 0.26 | 3.00 + 0.30 | 1.10‡ | 0.28 |
| Post (mean + SD) | 3.20 + 0.25 | 3.14 + 0.25 | 1.03‡ | 0.31 |
| Difference (mean + SD) | 0.13 + 0.10 | 0.14 + 0.15 | -0.43‡ | 0.67 |
| **Lens thickness (mm)** |  |  |  |  |
| Pre (mean + SD) | 3.38 + 0.25 | 3.59 + 0.25 | -3.80‡ | <0.001* |
| Post (median + IQR) | 3.27 + 0.41 | 3.50 + 0.31 | 496.50† | 0.003* |
| Difference (median + IQR) | -0.07 + 0.18 | -0.11 + 0.15 | 678.50† | 0.24 |

*TCPD*  Trabecular ciliary process distance; *ACD* Anterior chamber depth; *IQR* Interquartile range

† Mann- Whitney U test was conducted

‡ Independent – samples t test was conducted

* Statistically significant *P* < 0.05
