# Supplementary material for: Ultrasound biomicroscopy study of accommodative state in Smartphone abusers
Source: BMC Ophthalmol. 2022 Aug 3;22:330. doi: 10.1186/s12886-022-02557-x (PMC9347154; doi:10.1186/s12886-022-02557-x)
Supplement: Supplementary file 4 — Additional file 4: Supplementary Table 3. Correlation between Anterior chamber angle (ACA), spasm of accommodation and Smartphones spent hours. [file 12886_2022_2557_MOESM4_ESM.doc]

**Supplementary Table 3 Correlation between Anterior chamber angle (ACA) , spasm of accommodation and Smartphones spent hours**

|  | **Smartphones spent Hours (n = 40)** | ***P*-value** |
| --- | --- | --- |
| **Spasm of Accommodation** | 0.04† | 0.79 |
| **Superior ACA (degree)** |  |  |
| Pre | - 0.07‡ | 0.66 |
| Post | 0.12‡ | 0.46 |
| Difference | 0.25‡ | 0.13 |
| **Inferior ACA (degree)** |  |  |
| Pre | - 0,07‡ | 0.65 |
| Post | -0.04‡ | 0.81 |
| Difference | - 0.07‡ | 0.67 |
| **Temporal ACA (degree)** |  |  |
| Pre | - 0.15‡ | 0.37 |
| Post | - 0.14‡ | 0.40 |
| Difference | - 0.03‡ | 0.86 |
| **Nasal ACA (degree)** |  |  |
| Pre | - 0.07‡ | 0.89 |
| Post | - 0.13‡ | 0.42 |
| Difference | - 0.05‡ | 0.75 |

*ACA* Anterior chamber angle.

† Point-biserial correlation was conducted.

‡ Spearman’s correlation was conducted.

Significance level *P* < 0.05.
