# Supplementary material for: Ultrasound biomicroscopy study of accommodative state in Smartphone abusers
Source: BMC Ophthalmol. 2022 Aug 3;22:330. doi: 10.1186/s12886-022-02557-x (PMC9347154; doi:10.1186/s12886-022-02557-x)
Supplement: Supplementary file 5 — Additional file 5: Supplementary Table 4. Correlation between Anterior chamber depth (ACD), lens thickness, trabecular ciliary process distance (TCPD) and Smartphones spent hours. [file 12886_2022_2557_MOESM5_ESM.doc]

**Supplementary Table 4 Correlation between Anterior chamber depth (ACD), lens thickness, trabecular ciliary process distance (TCPD) and Smartphones spent hours**

|  | **Smartphones spent hours (n = 40)** | ***P*-value** |
| --- | --- | --- |
| **TCPD** |  |  |
| Pre | - 0.06‡ | 0.74 |
| Post | - 0.02‡ | 0.90 |
| Difference | 0.1‡ | 0.54 |
| **ACD** |  |  |
| Pre | - 0.03‡ | 0.87 |
| Post | - 0.15‡ | 0.36 |
| Difference | - 0.14‡ | 0.39 |
| **Lens thickness** |  |  |
| Pre | 0.13‡ | 0.43 |
| Post | 0.05‡ | 0.75 |
| Difference | - 0.04‡ | 0.80 |

*TCPD*  Trabecular ciliary process distance; *ACD* Anterior chamber depth

‡ Spearman’s correlation

Significance level *P* < 0.05
